# Failure of Nonoperative Treatment of a Vancouver B2 Periprosthetic Fracture About an Antibiotic Spacer

**DOI:** 10.7759/cureus.31028

**Published:** 2022-11-02

**Authors:** Nicholas Bertha, Lucas Nikkel

**Affiliations:** 1 Orthopedics, Penn State Health Milton S. Hershey Medical Center, Hershey, USA; 2 Orthopedics, Johns Hopkins Health System, Columbia, USA

**Keywords:** proximal femur fracture, total hip arthroplasty: tha, conversion to tha, vancouver classification, antibiotic spacer, periprosthetic hip fracture

## Abstract

Few case reports have been published describing the management of periprosthetic femur fractures with an antibiotic spacer. We report a case of a 58-year-old female who sustained a Vancouver B2 femur fracture with an antibiotic spacer that was initially treated with nonoperative measures. However, she went on to have increased fracture displacement and subsidence warranting operative intervention in the form of conversion to total hip arthroplasty with a diaphyseal fitting spline tapered stem and open reduction and internal fixation of the femur. We suggest that periprosthetic fractures about antibiotic spacers may be at a higher risk of displacement due to a lack of stem ingrowth potential and that early operative management may be more appropriate for these patients.

## Introduction

Periprosthetic fracture after total hip arthroplasty (THA) can occur intraoperatively, in the early perioperative period, or late. The overall rate of periprosthetic hip fractures is approximately 4% in primary THA and 6% in revision THA [1]. Periprosthetic fractures create a large economic burden, costing hundreds of millions of dollars to our healthcare system and significantly increasing hospital stays for patients [2-4].

Several classification systems are used to describe periprosthetic hip fractures, with the Vancouver classification system being the most widely used [5]. Review studies suggest that most Vancouver B2 and B3 benefit from surgical intervention with good outcomes [6]. There are, however, some studies describing or supporting attempts at non-surgical management for certain periprosthetic hip fractures [7].

In the setting of periprosthetic hip fracture and concomitant prosthetic hip joint infection, there are few studies to guide management. Here, we present a case report of a failed attempt at non-surgical management of a Vancouver B2 periprosthetic femur fracture about a PROSTALAC spacer.

## Case presentation

Patient information, clinical assessment, and further diagnostic assessment

**Figure 1:**
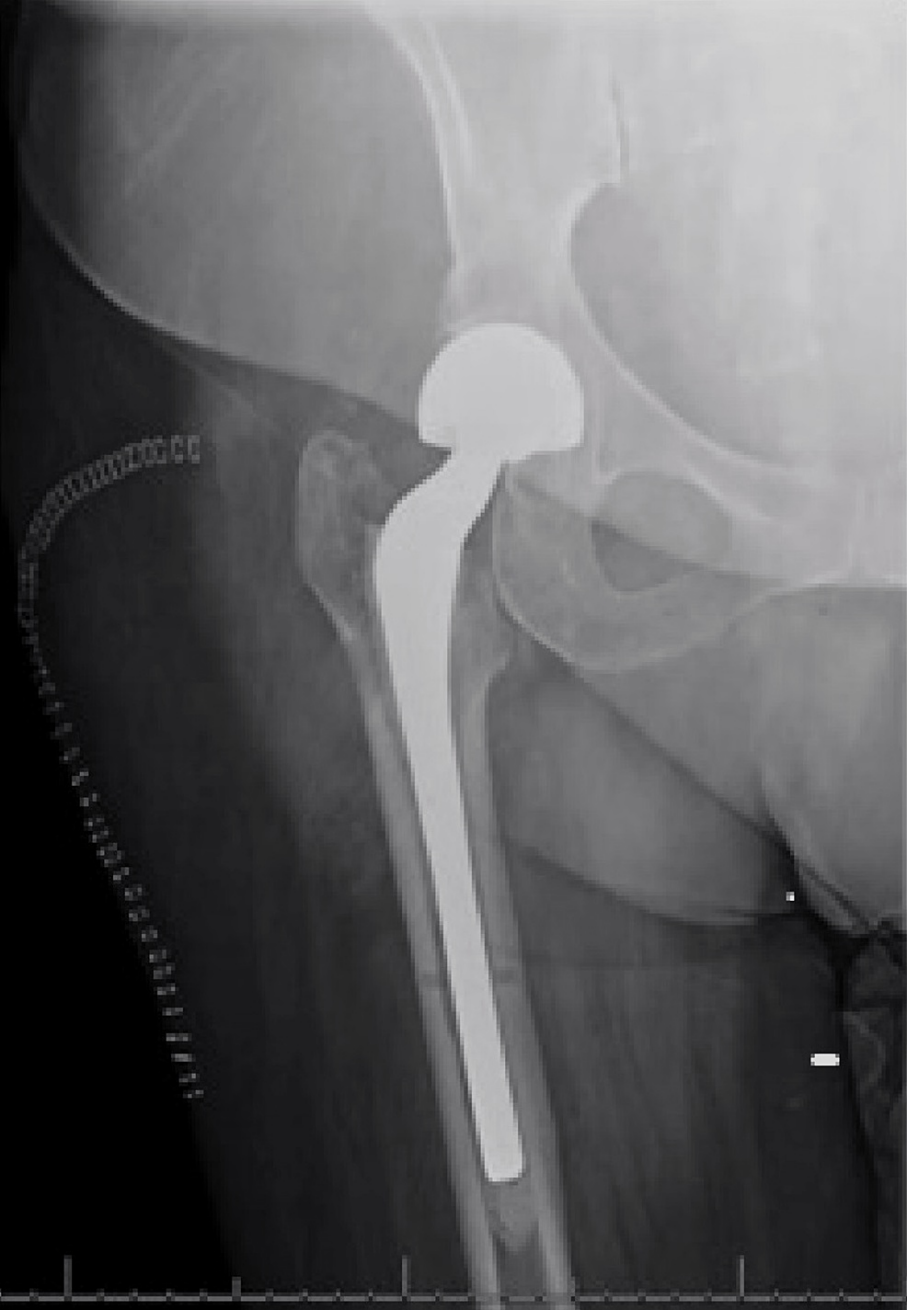
AP radiograph of the right hip one day prior to having increased hip pain shows the PROSTLALAC spacer in place without any evidence of loosening or acute fracture. AP - anterior-to-posterior

A 58-year-old female with a history of hypertension, diabetes, and hypothyroidism sustained a right intertrochanteric femur fracture (Arbeitsgemeinschaft für Osteosynthesefragen classification 31-A1) after a fall from standing and was treated at an outside institution with a trochanteric fixation nail. Approximately three weeks later, she developed purulent discharge and returned to the operating room for irrigation and debridement, with intraoperative cultures notable for methicillin-sensitive Staphylococcus aureus. She was treated with a four-week course of intravenous cefazolin and healed the wound, was ambulating without assistive devices and had minimal pain. Unfortunately, around eight months postoperatively she developed avascular necrosis of the femoral head, and given her prior infection, the trochanteric nail was removed and a PROSTALAC (Depuy, Warsaw, IN) hemiarthroplasty antibiotic spacer was implanted. No new labs or aspirate was obtained preoperatively. The patient initially did well clinically with no complications noted on an anterior-to-posterior (AP) radiograph (Figure 1).

**Figure 2:**
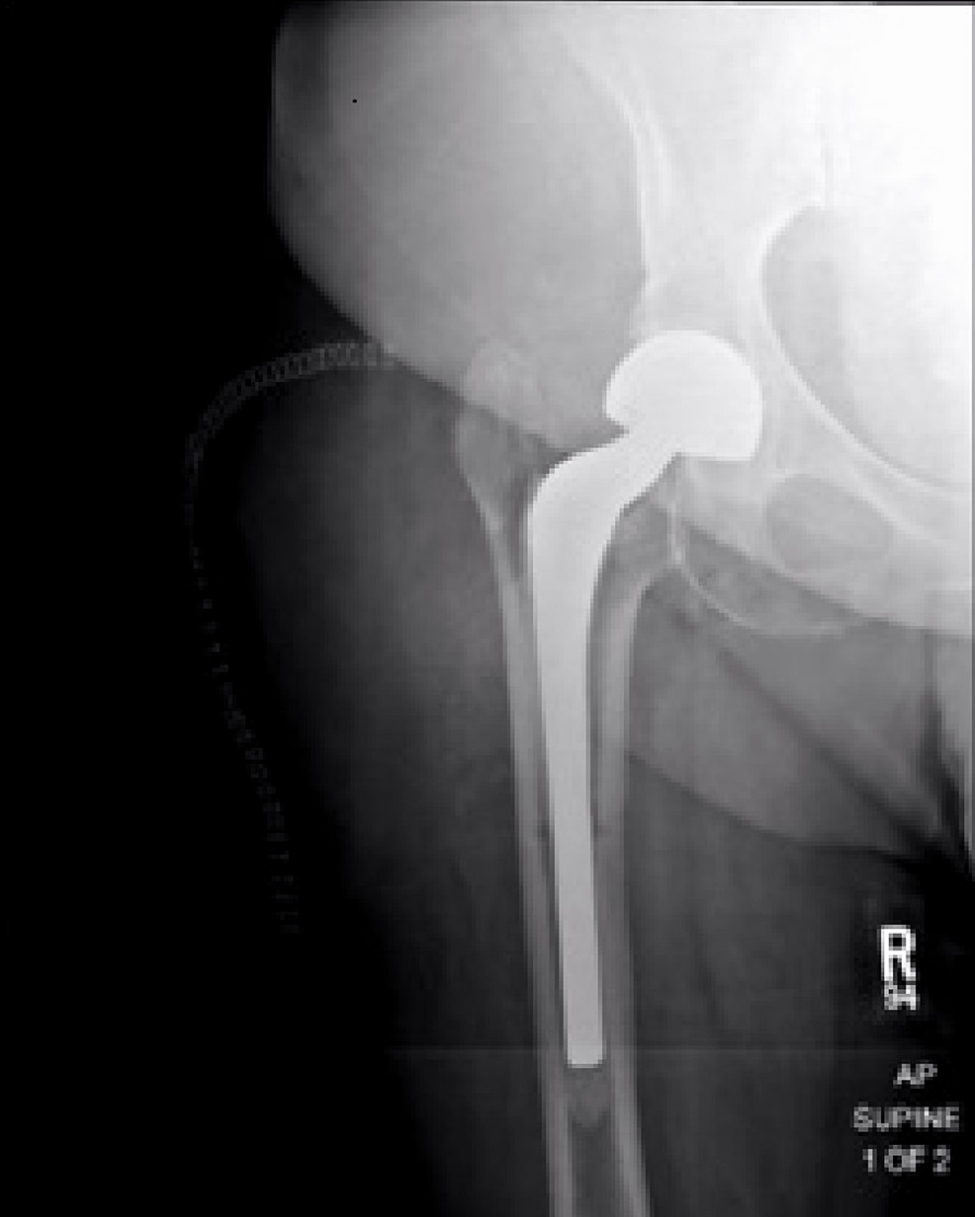
AP radiograph of the right hip after presenting with intractable hip pain two weeks status post placement of the PROSTALAC spacer There is concern for a periprosthetic femur fracture with subsidence of the femoral stem compared to the previous radiographs.

**Figure 3:**
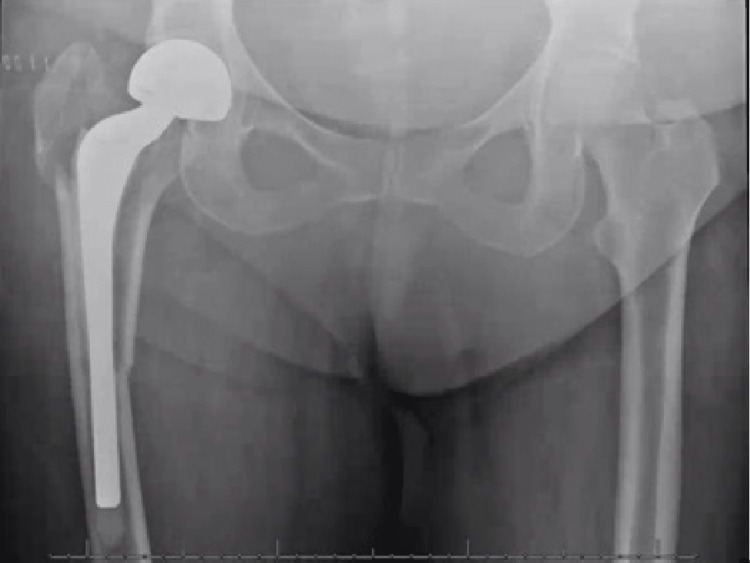
AP radiograph of the pelvis 12 days after presentation of intractable hip pain with continued inability to mobilize with therapy illustrates increased fracture displacement and femoral stem subsidence compared to the previous imaging.

The patient was admitted to our institution approximately two weeks postoperatively from placement of the hemiarthroplasty spacer with severe right hip pain after hearing a “snap” in her right hip. Clinically, her incisions were well healed and there were no signs of infection. Outside records showed final cultures from the spacer implantation procedure were negative for any microbial growth and the patient was not on any antibiotics. Radiographs showed a Vancouver B2 periprosthetic fracture with some femoral stem subsidence (Figure 2). The patient was made non-weight-bearing and planned to follow up with her original surgeon to determine a definitive treatment plan including the potential surgery. Unfortunately, the patient continued to have intractable pain and could not safely discharge to follow up with her surgeon. Twelve days after the presentation (approximately four weeks after placement of the antibiotic spacer), repeat radiographs were obtained and showed increased fracture displacement and further subsidence of the hemiarthroplasty spacer (Figure 3), at which point the arthroplasty service was asked to assist with management. The hip was aspirated and was notable for a synovial fluid nucleated cell count of 3,400 with 42% neutrophils and a negative gram stain. Cultures remained negative.

Therapeutic intervention

**Figure 4:**
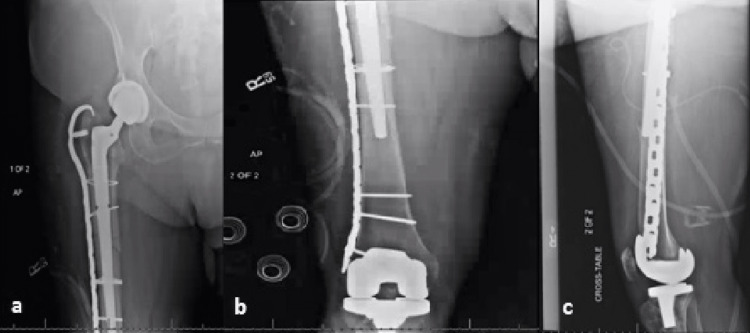
Postoperative radiographs including right proximal femur AP (a), distal femur AP (b), and lateral (c) views. Radiographs depict fracture fixation with a diaphyseal fitting tapered femoral stem, cerclage wires, and lateral trochanteric hook plate. Anterior cortical perforation can be visualized on the lateral radiograph.

Due to intractable pain and lack of ingrowth potential from the hemiarthroplasty spacer, the patient underwent conversion to THA with open reduction and internal fixation of the femur via a posterolateral surgical approach. There was no necrotic tissue and intraoperative histology was negative for acute inflammation on all frozen section specimens. Based on surgeon preference, a modular, diaphyseal engaging, splined, tapered stem was selected (Figures 4a-4c) with plan for adjunctive fixation with lateral submuscular femoral trochanteric hook plate. During reaming of the femoral canal an incidental anterior femoral shaft cortical perforation distal to the site of the fracture occurred which was addressed using a longer femoral trochanteric hook plate to bypass both the initial periprosthetic fracture as well as the anterior cortical perforation that occurred. A 10 French hemovac drain was placed, the wound was closed, and a negative pressure incisional vacuum dressing was applied to the wound.

Follow-up and outcome

**Figure 5:**
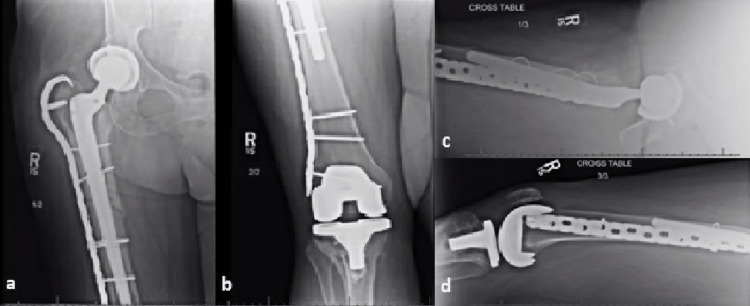
Three-month follow-up radiographs of the right hip including proximal femur AP (a), distal femur AP (b), proximal lateral (c), and distal lateral (d) views. Radiographs do not show any evidence of subsidence and the fracture remains stable.

Postoperatively, the patient’s weight-bearing was restricted, and she was maintained on oral antibiotics for three weeks. Intraoperative tissue cultures remained negative at fourteen days postoperatively. She was last seen at her three month follow up appointment at which time she was doing well with minimal pain, not showing any signs of infection, and had no evidence of complications on radiographs (Figures 5a-5d). She was advanced to weight bearing as tolerated at that time. She continued participating in physical therapy for the next few months to help regain her balance, however, she continues to walk with a walker. Patient was last seen approximately fifteen months postoperatively and reports she feels that she has a slight limp but is otherwise happy and performing all activities she would like.

## Discussion

Periprosthetic femur fractures represent a complex injury that creates multiple challenges. These challenges need to be managed differently when the fracture is about an antibiotic spacer as opposed to a porous coated stem. To our knowledge, this is the first case report of an attempt at the non-operative treatment of a Vancouver B2 periprosthetic fracture around an antibiotic-cement spacer. We believe that the fracture may have been related to a stress riser from the screw hole in the femur from the nail removal. Given early failure, it is recommended that these rare fractures be treated surgically and without unnecessary delay. In the setting of a Vancouver B2 fracture without evidence of infection, we would recommend revision of THA with a long femoral stem to bypass the fracture with open reduction internal fixation. Other options would include utilizing a fully cemented Exeter-style custom-made articulating spacer to perform a single-stage revision. We believe that if a preoperative infection workup was completed prior to PROSTALAC placement, then definitive fixation could have occurred at this point and potentially prevented the fracture from occurring.

There have been few studies describing attempted nonoperative treatment for Vancouver B1/B2 periprosthetic femur fractures. In Lee et al.’s study [7] describing nonoperative treatment for minimally displaced fractures around a cementless stem, they found a 100% union rate of Vancouver B1 fractures and a 75% union rate of Vancouver B2 fractures when treated non-operatively. Unlike the cementless stems analyzed in this study, our patient had an antibiotic spacer that did not allow ingrowth potential.

In the setting of a femur fracture with deep infection around a nail, the use of antibiotic-coated nails can be used to achieve fixation and manage the infection with adequate results [8]. These fractures are typical of the femoral diaphysis and can be stable with a nail alone. Furthermore, there is a case report regarding a Vancouver C fracture about a PROSTALAC spacer which was treated with an exchange of an antibiotic spacer and open reduction internal fixation with an antibiotic-coated plate. This patient did go on to achieve union and inflammatory markers returned to within normal limits [9].

Hip spacers generally have a medial-lateral wedge shape that with subsidence continues to exert displacing hoop stresses to the fractured bone. Therefore, sufficient stabilization of a fracture about one of these stems would be unlikely. We hypothesize that for this reason, periprosthetic fractures about a PROSTALAC spacer cannot be adequately treated nonoperatively, as there is no ingrowth potential to prevent implant subsidence in the setting of fracture. In this case, some of the greater trochanteric fragment displacement was likely related to the pull of the abductors and may have occurred with or without a stem with ingrowth potential.

In this case, concern for underlying infection led to the treatment of aseptic avascular necrosis with an antibiotic-coated hemiarthroplasty spacer. Ideally, a full preoperative workup, including inflammatory markers and hip aspiration, combined with intraoperative evaluation, would help guide the decision about whether to convert directly to THA or to place a temporary spacer. Had this patient met the musculoskeletal infection society (MSIS) criteria supporting a potential infection at either preoperative workup or after the fracture occurred, the appropriate treatment would include conversion to a longer antibiotic spacer, bypassing the fracture site, along with open reduction with internal fixation [10,11].

## Conclusions

While periprosthetic fractures about an antibiotic spacer are a rare complication, we believe that patients presenting with periprosthetic fractures about antibiotic spacers may be at a higher risk of worsening displacement when attempting non-operative treatment. Given this risk of displacement, we recommend treating these fractures operatively without unnecessary delay. If concern for infection persists, we recommend converting to a longer antibiotic spacer that bypasses the fracture along with open reduction internal fixation.
